# Research of the Changes in the Psychological Status of Chinese University Students and the Influencing Factors During the COVID-19 Pandemic

**DOI:** 10.3389/fpsyg.2022.891778

**Published:** 2022-05-31

**Authors:** Chen Liu, Jiayi Tang, Chao Shen, Xingya Zhan, Enhao Bu, Baozhen Shen, Wenhao Huang

**Affiliations:** ^1^School of Public Health, Xuzhou Medical University, Xuzhou, China; ^2^Key Laboratory of Infectious Diseases, Department of Epidemiology and Biostatistics, School of Public Health, Nanjing Medical University, Nanjing, China; ^3^School of Management, Xuzhou Medical University, Xuzhou, China

**Keywords:** COVID-19, psychological state, influencing factors, college students, questionnaire survey

## Abstract

**Background:**

Psychological dynamics of college students have changed during the COVID-19 outbreak but little research has been done in this area. The purpose of this study is to investigate the dynamic changes in the mental health status of college students since the outbreak of the COVID-19 pandemic 1 year and the influencing factors.

**Methods:**

The research period was from February 2020 to August 2021. 384 college students were analyzed three times during this period on the recognition and psychological state of the pandemic.

**Results:**

During the period from February 2020 to August 2021, in general, the positive scores rose from 20.79 to 23.46, while the negative scores dropped from 17.41 to 14.00. The regression analysis results on the influencing factors showed the degree of recognition of the pandemic is all significant in the three phases (*p* < 0.05).

**Conclusion:**

With the effective control of the pandemic, the mental state of the students showed a slight improvement in the environment of sporadic cases. Behavior has a partial mediating effect between the source of fear and psychological changes. Correct behavior guidance can effectively reduce the psychological changes caused by college students’ fear.

## Introduction

The sudden attack of the COVID-19 pandemic has drastic negative effects on every segment of human society in the socio-psychological and physical paradigm. The sustainable development goals in all aspects of the global economy, agriculture, environment and education were materially affected (Wang and Huang, 2021). In February 2020, governments of various countries implemented various measures such as city blockade, travel warning regulations, and home medical observation to prevent and control the spread of the virus. Especially in China, until 2021, the lockdown policy is still implemented in areas with more cases. These policies restricted people’s travel and changed their lifestyles. The implementation had a serious impact on the mental state of people around the world.

In March 2022, the World Health Organization (WHO) said that the global prevalence of anxiety and depression increased by 25% in the first year of the COVID-19 epidemic. Among them, according to the CDC’s COVID-19 Data from NCHS—Mental Health: Household Pulse Survey, the COVID-19 pandemic has increased the prevalence of mental illness, including depression, anxiety, and suicide rates in the United States (Centers for Disease Control and Prevention, 2020). Similarly, a survey of 2,400 Japanese people by Kikuchi et al. (2020) showed that the mental health of Japanese people deteriorated from the early stage of COVID-19 to the stage of community transmission. China is currently one of the countries with the strictest implementation of various measures, such as city blockade, travel warning regulations and home medical observation, also one of the countries with the best epidemic control. A large sample survey conducted by Chinese scholars across the country showed that 35% of the public experienced psychological distress during the outbreak of COVID-19 (Qiu et al., 2020). And studies have reported various psychological problems, such as anxiety, fear, depression, and insomnia in the public during the epidemic (Li W. et al., 2020). The negative effects that the pandemics incurred are drastic.

Mental health is an important part of physical and mental health, and it is also an indispensable important content in college students’ study, growth and life. The state of college students’ mental health directly determines the outcome of college education and the development prospects of the students themselves (GS, 2019). Although the cure rate of COVID-19 has been greatly increased, the public is prone to anxiety and panic due to its extremely contagious nature, susceptibility to mutation and lack of rapid treatment methods. The comprehensive awareness of COVID-19 is generally low among college students (Agorastos et al., 2021). Thus, they are more prone to psychological problems. In a COVID-19 study on youth conducted, 12.8% of participants were diagnosed with PTSD, which shows the significance of public health emergencies on the youth population (Liang et al., 2020). Meanwhile, the United States CDC found that mood and anxiety disorders tripled in 2020 compared with 2019, especially among 18–29-year-olds (Centers for Disease Control and Prevention, 2020). Moreover, a study by Chinese scholars showed that among 3,881 ordinary Chinese college students, the incidence of anxiety disorders was 26.60% (Chang et al., 2020). Sun et al. (2021) found that 1,921 Chinese college students facing quarantine reported more common psychiatric symptoms.

In this context, this work is devoted to exploring the changes and influencing factors of the psychological state of Chinese college students since the COVID-19 pandemic began one and a half years ago. Since college students are considered to be the workhorses of the future society, their mental state during the pandemic should also be taken seriously. This is significant for major Chinese universities to take correct intervention measures for the problems of college students’ psychological state, even for universities in countries other than China.

## Literature Review

In terms of the overall psychology of Chinese citizens affected by the pandemic, domestic scholars have paid more attention to the personal factors related to the difference in the degree of the pandemic affecting mental health including age, gender, place of residence, whether they are close contacts and physical health (Hossain et al., 2020). Studies have shown that women and students, whether have symptoms of the COVID-19, pandemic-related news, social media connections, psychosocial support, and trust in the government may all affect the public’s mental state (Huang et al., 2020; Yaner, 2020). Among them, the public’s trust in the government can significantly alleviate the public’s psychological crisis (Yaner, 2020), and good social support can also alleviate public anxiety and depression, making them more positive and optimistic in responding to public emergencies (Lei and Fei, 2020). In addition, during the COVID-19 pandemic, the public’s environment will affect their psychological state. Studies have shown that with the increase in the cumulative number of confirmed local cases and the severity of the epidemic, the public’s psychology tends to be tense and negative (Fang and Bing, 2020; Xu and Tingting, 2020; Haijuan and Ziming, 2021). Regarding college students, the related factors affecting the mental health of college students include only-child status, marital status, family economic status, physical and mental health status (Haijuan and Ziming, 2021). Among them, the anxiety and depression of the undergraduate group are more serious than the postgraduate group; the only child is more serious than the non-only child; the college students with difficult family financial status and poor physical are more serious. There are also studies showing that seniors experience more anxiety than freshmen (Li et al., 2021). Besides, worldwide phenomena have established risk factors for the mental health and well-being of youth over the past 15 years, such as the economic crisis, climate change and the COVID-19 pandemic (Poletti et al., 2022). The burden of their cumulative impact on mental health may be more severe, especially in the impact of psychopathological manifestations. Furthermore, the outbreak of COVID-19 will affect the development of the environment and economy. The outbreak of COVID-19 has improved air quality in China in the short term. However, in the long run, air quality may deteriorate beyond pre-event levels when China fully lifts its lockdown and resumes large-scale industrial production (Wang and Su, 2020). The COVID-19 pandemic has triggered economic and energy crises, and China will play an important role in the post-pandemic global economic recovery effect (Wang et al., 2021). Therefore, whether it is the pandemic itself or the environmental and economic changes caused by the pandemic, it may become a risk factor affecting the psychological state of college students.

Developed countries aim to study the sustainability of education while developing countries pay more attention to economic sustainability during the pandemic (Wang and Huang, 2021). Compared with research on economic development, there is a lack of research on the impact of the pandemic on education in China, and research related to mental health education is even scarcer. Besides, many existing studies have focused on the mental health status of the population, most have focused on the general population or health care workers, so the results may not be suitable for college students (Chen et al., 2020; Gao et al., 2020; Feng and Zhang, 2021). During the pandemic, there is still little research on whether the general public’s mental state characteristics are suitable for college students. Meanwhile, in the current research on college students, the research on influencing factors often ignores general information such as gender, ethnicity and region, but focuses on their identities as students and children. What’s more, the selection of samples is often limited, such as confined to a certain university, a certain major, etc. Moreover, most of the current research conducts horizontal surveys at the same time (Wise et al., 2020). Few researchers conduct longitudinal surveys of college students’ mental health in stages.

Thus, based on the existing literature, this study changes the selection of influencing factors and selects the general information of college students and the characteristic information of their identity as the variables. At the same time, the social environment, namely the development of the pandemic, is the most important variable in comprehensively examining the influence of personal and social factors on the mental health of college students. Our innovation is to conduct a longitudinal survey of college students’ mental state in three stages within one and a half years. In conclusion, this work has contributed to the existing literature in the following two aspects. First, the dynamic changes in the mental state of the college student population with the normalization of the COVID-19 pandemic appear to be particularly important, but there are few studies in this area. Our research fills this gap and can predict future development based on the dynamic trend of changes in the psychological state of college students. Second, the research on the influencing factors in this paper can give suggestions to colleges and universities to implement measures to improve the psychological state of college students.

## Materials and Methods

### Participants

The participants of the survey are college students in China, including five colleges and over 20 classes around Xuzhou, Jiangsu Province. All participants were randomly recruited and voluntarily participated. They wrote informed consent before completing the study questionnaire.

### Survey Period

This survey period was conducted in three phases. The first phase of the survey is from February to March 2020, the second phase is from August to September 2020, and the third phase is from July to August 2021. The reason for choosing the first period is that March 2020 was the first month when the Chinese government activated level-1 public health emergency responses in 31 provincial-level regions in mainland China (Huang et al., 2020). The second phase of August to September 2020 is selected because of Joint Prevention and Control Mechanism of the State Council issued the “Guiding Opinions on Doing a Good Job in the Normalization of the Prevention and Control of the COVID-19 pandemic” on May 7, 2020. Which mentioned that the pandemic has entered the period of normalization of prevention and control at this time, and the students who were surveyed from August to September returned to school, so that we can conduct the questionnaire survey. The reason why the last phase chose August 2021 is the pandemic situation in Nanjing, Chengdu, and other places became serious in July 2021. There were sporadic cases of the pandemic, which may have an impact on the mental health of students.

### Investigation

In the first phase of the survey, the participants were contacted by the counselors of universities and issued questionnaires to the classes they managed. The monitor of each class will ask participants to take screenshots of their mobile phones at the end of the survey to prove that the students in the class have completed the survey. Then we count the number of completions and record the identity of the participants. We collected information on the number of participants from various counselors and teachers, continued to distribute the questionnaires to the students who had filled in the questionnaires in the second and third phases of the survey, and urged them to complete the questionnaires to ensure that this longitudinal survey was all from the same students’ reply. The first phase received 403 questionnaires, the second phase received 384 questionnaires, and the third phase received 385 questionnaires. We finally obtained 384 valid samples, which are guaranteed to be from the same student’s responses at different times, after excluding some question samples that failed to participate in the survey three times.

### Research Tool

We conduct research by distributing questionnaires. All students receive surveys on general information, recognition of the epidemic, sources of fear, mental health, and behavior change. (1) General information: gender, ethnicity, major, grade, and so on; (2) Recognition of the pandemic: the degree of recognition of the pandemic, the preventive measures of COVID-19, and so on; (3) Source of fear: fear of a highly contagious virus, fear of no specific treatment, and so on; (4) Mental health survey: generally happy, feel the interesting daily life, and so on; (5) Behavior change survey: pay attention to disinfection and wash hands frequently, and so on. The contents of the 2, 3, 4, and 5 parts are based on the “SARS Social Psychological Survey Questionnaire (National Version)” developed and compiled by the Institute of Psychology of the Chinese Academy of Sciences, combined with the COVID-19 pandemic, based on the original scale and made corresponding additions and deletions. The main part of the questionnaire uses the Likert five-level scale to score points. In the “mental states” part, positive mental states are counted as positive points, and negative mental states are counted as negative points (Chuanfeng and Jianwen, 2003).

The main psychometric indicators included in our original questionnaire have two aspects: positive psychological state and negative psychological state. The positive psychological state includes generally happy, feeling interesting in daily life, being able to face problems bravely, focusing on doing things, I feel that I can make up my mind when doing things and feeling energetic; the negative psychological state includes feeling depressed and unhappy, feeling mentally stressed, feeling unable to overcome difficulties, feeling more emotional, feeling useless and insomnia because of worry. These were developed by the Institute of Psychology, the Chinese Academy of Sciences.

For the validity and reliability of the survey, the results of confirmatory factor analysis (CFA) of the source of fear survey showed that: χ^2^/df = 4.229 (<5.0), root mean square error of approximate (RMSEA) = 0.070, normal of fit index (NFI) = 0.961 (>0.9), incremental fitting index (IFI) = 0.970 (>0.9) and comparative fit index (CFI) = 0.970 (>0.9). The results of Confirmatory Factor Analysis of other surveys also meet the requirements, which means that the validity of the original survey is good. Furthermore, the Cronbach’s alpha for internal consistency for the use of our modified SARS Social Psychological Survey Questionnaire (National Version) was 0.907, which verifies the reliability of the survey.

### Statistical Analysis

In the data processing in this article, the quantitative data is expressed by *M* (P25, P75) on the psychological state score scale, fear source identification score scale and the behavior change score scale; qualitative data is expressed by the case (%); comparison between groups is expressed by Kruskal–Wallis *H* test; the influencing factors of students’ psychological changes adopt hierarchical multiple regression analysis. All statistical analyses were performed using SPSS 23.0 for Windows (IBM, Somers, NY, United States). The structural equation model was constructed using AMOS 23.0 software (IBM, Somers, NY, United States) to verify the relationship between variables and the mediation effect, and the Bootstrap method was used to test the mediating effect. The difference is statistically significant when *p* < 0.05.

## Results

### Demographic Characteristics

The demographic characteristics of all subjects are summarized in Table 1. The number of valid questionnaires returned was 384, the main population distribution was 142 males, accounting for 37%, 242 females, accounting for 63%; 359 Han nationalities, accounting for 93.5%, 25 non-Han nationalities, accounting for 6.5%; 261 medical students, accounting for 68%, 123 students from other majors, accounting for 32%; 282 first and second-grade students, accounting for 73.5%, 102 third to fifth-grade students, accounting for 26.6%; 182 student leaders, accounting for 47.4%, non-student leaders 202 people, accounting for 52.6%.

**TABLE 1:** Demographic characteristics of participants.

Variables	*N*	Proportion (%)
**Gender**		
Male	142	37
Female	242	63
**Ethnicity**		
Han nationality	359	93.5
Non-Han nationality	25	6.5
**Major**		
Medical major	261	68
Non-medical major	123	32
**Grade**		
1	165	43
2	117	30.5
3	46	12
4	55	14.3
5	1	0.3
**Whether a student cadre**		
Yes	182	47.4
No	202	52.6

### Mental State Scale Score

As shown in Tables 2, 3, during the entire survey period, from the perspective of positive indicators (the larger the positive indicator, the better the mental state of the people). The respondents agree with most indicators to a higher degree: they feel that they are happy, able to concentrate, etc. It can be seen that the mentality of college students is more inclined to be optimistic. From the first phase to the second phase, the positive index rises quickly, but the second phase and the third phase rise are not obvious, the negative index correspondingly declines. Besides, generally happy, feel the interesting in daily life, able to face problems bravely, focus on doing things, feel that I can make up my mind when doing things, feel energetic, project total score, feel depressed and unhappy, feel mentally stressed, feel unable to overcome difficulties, feel more emotional, feel useless, insomnia because of worry, and project total score showed significant differences among three stages (*p* < 0.05).

**TABLE 2:** Score of college students’ positive mental state scale and Kruskal–Wallis *H* test in the three stages of the pandemic [Points, *M* (P25, P75)].

Project	Phase 1	Phase 2	Phase 3	*H*-value	*p*-value
Generally happy	4 (3, 4)	4 (3, 5)	4 (4, 5)	12.855	0.002
Feel the interesting in daily life	4 (3, 4)	4 (3, 4)	4 (3, 5)	17.941	<0.001
Able to face problems bravely	4 (3, 4)	4 (4, 5)	4 (4, 5)	13.400	0.001
Focus on doing things	4 (3, 4)	4 (3, 4)	4 (3, 4)	10.635	0.005
I feel that I can make up my mind when doing things	4 (3, 4)	4 (3, 4)	4 (3, 5)	18.744	<0.001
Feel energetic	4 (3, 4)	4 (3, 4)	4 (3, 4)	23.267	<0.001
Project total score	22 (19, 25)	23 (20, 25)	24 (20, 27)	22.357	<0.001

**TABLE 3:** Score of college students’ negative mental state scale and Kruskal–Wallis *H* test in the three stages of the pandemic [Points, *M* (P25, P75)].

Project	Phase 1	Phase 2	Phase 3	*H*-value	*p-*value
Feel depressed and unhappy	3 (2, 4)	2 (2, 3)	2 (2, 3)	34.046	<0.001
Feel mentally stressed	3 (2, 4)	2 (2, 3)	2 (2, 3)	65.122	<0.001
Feel unable to overcome difficulties	3 (2, 4)	2 (2, 3)	2 (2, 3)	54.618	<0.001
Feel more emotional	3 (2, 4)	2 (2, 3)	2 (2, 3)	69.400	<0.001
Feel useless	3 (2, 4)	2 (2, 3)	2 (1, 3)	50.225	<0.001
Insomnia because of worry	3 (2, 4)	2 (1, 3)	2 (1, 3)	85.467	<0.001
Project total score	17.5 (13, 22)	14 (12, 17)	14 (11, 18)	88.848	<0.001

*State frequency values 1-3-5, representing “never”-“sometimes”-“frequently” and other different degrees; other numerical meanings are between these situations.*

### Hierarchical Multiple Regression Analysis

Take Gender, Ethnicity, Grade, Major, whether a student leader as control variables, recognition of the pandemic, fear of a highly contagious virus, fear of no specific treatment, fear of the emergence of sporadic cases as independent variables. The total mental state score of the first stage, the total mental state score of the second stage, and the total mental state score of the third stage were included in the hierarchical linear regression as the dependent variable. As shown in Table 4, the mental health status of this group of college students has different influencing factors in different periods. Among them, ethnicity, major, and the recognition of the pandemic directly affect their mental health in the first stage; the direct influencing factors in the second stage are grade, the recognition of the pandemic, and the fear of infectiousness of the virus; and the direct influencing factors in the third stage are ethnicity, grade, and degree of recognition about the pandemic. The independent variable explains the dependent variable 22.4, 10.8, and 8.6% variation. Besides, there is no multicollinearity between the data (Supplementary Table 1).

**TABLE 4:** Hierarchical multiple regression analysis of influencing factors of college students’ psychological changes.

Variable	Dependent variable: Total mental state score		
	Phase 1	Phase 2	Phase 3
**Controlled variable**			
Gender	0.039	–0.029	0.021
Ethnicity	−0.103*	–0.006	−0.119*
Grade	–0.014	−0.143**	−0.173***
Major	0.214***	–0.099	0.030
Whether a student leader	0.073	–0.062	–0.018
**Independent variable**			
Recognition to the pandemic	−0.180***	−0.114*	−0.174***
Fear of a highly contagious virus	0.250***	0.150*	0.083
Fear of no specific treatment	–0.041	0.32	–0.056
Fear of the emergence of sporadic cases	0.068	0.095	–0.032
*R* ^2^	0.224	0.108	0.086
*F*	12.565***	5.028***	3.915**
Δ*R*^2^	0.070	0.043	0.043
Δ*F*	7.044***	3.570**	3.525**

*p < 0.05, **p < 0.01, ***p < 0.001.

### Fear Source Identification Score Scale, and the Behavior Change Score Scale

As shown in Table 5, among the five sources of college students’ fear of the pandemic, only “Negative news from the web” showed significant differences among the three stages (*p* < 0.05). As shown in Table 6, “No cold symptoms but also take some drugs to prevent it,” “Start eating heavily to relieve your emotions,” “Do what you usually have no time to do,” “Smoking and drinking are even more popular than usual,” and “Pray for ancestors or gods,” the differences in the scores of the three stages were statistically significant (*p* < 0.05).

**TABLE 5:** The Kruskal–Wallis *H* test scores of college students’ identification with the source of fear in three stages of the pandemic [Points, *M* (P25, P75)].

Project	Phase 1	Phase 2	Phase 3	*H*-value	*p*-value
Viral infectivity is strong	4 (3, 5)	4 (4, 5)	4 (4, 5)	4.259	0.119
The virus could lead to death	4 (3, 4)	4 (3, 5)	4 (3, 5)	4.724	0.094
There is no quick and effective treatment available yet	4 (3, 4)	4 (3, 4)	4 (3, 4)	5.793	0.055
The increase in confirmed diagnoses is widely reported	4 (3, 4)	4 (3, 4)	4 (3, 4)	2.790	0.248
Negative news from the web	3 (3, 4)	3 (2, 4)	3 (2, 4)	9.327	0.009

**TABLE 6:** Behavior change scale for college students and Kruskal–Wallis *H* test score in three stages of pandemic [Points, *M* (P25, P75)].

Project	Phase 1	Phase 2	Phase 3	*H*-value	*p*-value
Pay attention to disinfection and wash hands frequently	4 (3, 5)	4 (3, 5)	4 (3, 5)	0.695	0.706
Minimize contact with others	4 (3, 5)	4 (3, 5)	4 (3, 5)	2.534	0.282
More concerned about the media release than usual	4 (3, 5)	4 (3, 5)	4 (3, 5)	1.404	0.496
Provide people around you with ways to prevent the pandemic	4 (3, 5)	4 (3, 5)	4 (3, 5)	0.752	0.687
No cold symptoms but also take some drugs to prevent it	3 (2, 4)	2 (1, 3)	2 (1, 3)	45.911	<0.001
Start eating heavily to relieve emotions	2 (2, 4)	2 (1, 2)	2 (1, 2)	90.908	<0.001
Do what you usually have no time to do	3 (3, 4)	3 (2, 4)	3 (2, 4)	65.867	<0.001
Smoking and drinking are even more popular than usual	2 (1, 4)	1 (1, 2)	1 (1, 2)	106.284	<0.001
Pray for ancestors or gods	2 (1, 4)	1 (1, 2)	1 (1, 2)	96.446	<0.001

### Establishment of the Mediating Effect Model

**FIGURE 1:**
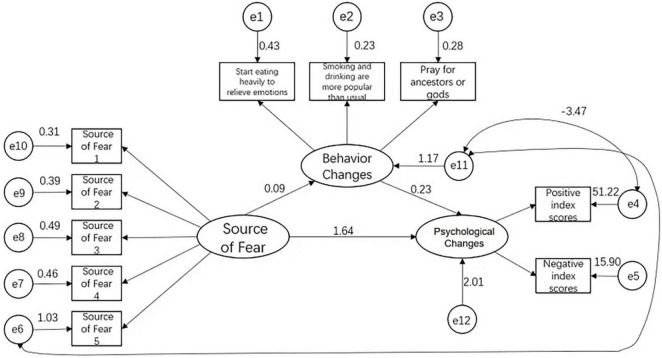
The path analysis obtained from the structural equation model (SEM) of the relationships from the results of the source of fear, behavior changes, and psychological changes.

Using AMOS 23.0 software, using the source of fear as the independent variable, behavioral change as the mediator, and psychological change as the dependent variable, a structural equation model was established, and the Maximum Likelihood Estimate was used to modify and fit the model to test the hypothesis. The model fit results showed: χ^2^/df = 2.013, root mean square error of approximate (RMSEA) = 0.077, goodness-of-fit index (GFI) = 0.953 (>0.9), adjusted goodness-of-fit index (AGFI) = 0.928 (>0.9), normal of fit index (NFI) = 0.943 (>0.9), incremental fitting index (IFI) = 0.950 (>0.9), comparative fit index (CFI) = 0.950 (>0.9), which means the model fits well, and each path coefficient is statistically significant (*p* < 0.05), indicating that the mediating effect test is feasible. The final fitting model and standardized path coefficients are shown in Figure 1. The Bias-Corrected Bootstrap method was further used to test the significance of the mediating effect. Repeated sampling was repeated 5,000 times. The results showed that the 95% CI of the direct and indirect effects of the source of fear and psychological changes does not include 0 (*p* < 0.05). The mediating effect is significant, indicating that the behavioral performance has a partial mediating effect between the source of fear and psychological changes. See Table 7.

**TABLE 7:** Mediating effect.

Path	Effect size	Standard error	95% CI	*p*-value
Mediating effect	0.022	0.018	0.000, 0.117	0.045
Direct effect	1.642	0.264	1.133, 2.154	0.000
Total effect	1.642	0.266	1.150, 2.178	0.000

## Discussion

Different individuals have different personality tendencies, physical conditions, social identities, and risk perception abilities, which makes their impact on the COVID-19 pandemic have individual differences. This study believes that recognition of the pandemic is an important factor that significantly affects the psychological state of college students in all three stages. In the early stage of the outbreak, although the number of confirmed cases continued to rise, more positive news came along with the progress of virus research and the increase in discharge cure rates, which can affect the psychological state of students. According to recent research, the key to China’s success in avoiding a second wave of the COVID-19 pandemic is the large-scale integration of digital technology and public health without hesitation (Wang et al., 2021). Through this college students can receive COVID-19-related information such as the number of confirmed cases and the activity range of confirmed persons, to improve their recognition of the pandemic. On the one hand, cognitive appraisals were differentially related to negative emotion, positive emotion and sleep problems (Li J. B. et al., 2020). In the cultural environment of China, people’s behavior is easy to follow “feeling,” which determines that perception and belief play an important role in their behavior selection. Therefore, it is very important to correct the wrong “feeling” of college students. We need to use reasonable strategies to let them know the correct information and get rid of false beliefs and perceptions. On the other hand, Muzi et al. (2021) found that teenagers during pandemics showed more social media use problems than in pre-pandemic samples. Besides, studies have found that frequent use of social media during the pandemic can lead to information overload and over concern among individuals, which may increase the negative emotions of college students (Farooq et al., 2020; Muzi et al., 2021). Therefore, ignorantly encouraging students to pay attention to the pandemic-related information on the Internet is not a wise strategy for college students, but should reduce information overload via the clear structuring and communication of reliable health information.

The mental state of non-Han minority students is worse than that of Han students. First, in recent years, more and more ethnic minority college students have entered university gates. Due to differences in ethnic culture, language, and living habits, it is not very easy for ethnic minority students to adapt to a new environment in a short period. At the same time, this is a huge process of physical and psychological transformation for them (Haiyi, 2017). Second, some ethnic minority students lack good self-regulation ability and are more likely to have different degrees of psychological problems than Han students. There is currently a lack of research on the psychological state of Chinese ethnic minority college students during the pandemic. However, the research by Trammell found that Latinx and Asian students experienced higher COVID-19-related threats and negative beliefs than White students. Asian students experienced more discrimination (Trammell et al., 2021). The results showed that the psychosocial impacts of a pandemic on students vary by race/ethnicity.

Major is also a factor that affects the psychological state of college students. The survey results show that medical students have better mental health status than non-medical students. It may be because medical students can use the professional knowledge they have learned to distinguish the authenticity of online news. They have more understanding of modern medical technology and level, and national health policies, so they will have fewer negative emotions such as fear and anxiety. An Israeli survey shows that the high overall satisfaction rate with online learning along with low technical difficulties was closely correlated to the desire to continue online learning (*p* < 0.01) (Sandhaus et al., 2020). Satisfaction with online learning may also improve the mental state of college students. But another American survey found that a total of 741 medical students (74.7%) believed that the pandemic had seriously disrupted their medical education, since the beginning of the pandemic, self-reported emotional exhaustion and burnout have increased statistically significantly (*p* < 0.001) (Harries et al., 2021). Another study of 549 medical students found that 341 (62.3%), 410 (74.6%), 344 (62.6%), and 379 (69%) had self-reported anxiety, depression, insomnia, and distress (Essangri et al., 2021). The reason why the above survey contradicts our research results may be that most of our participants are freshmen to juniors, and few students have already participated in clinical work or are facing clinical offline courses, so they do not feel anxious about such courses. It may also be because the students are not professionally mature enough to deal with one of the most serious public health crises in the world (Zheng et al., 2021).

It can also be concluded from Table 1 that the development and changes of the pandemic have a significant impact on the psychological state of college students. First of all, because the development of the pandemic is uncontrollable and uncertain for the masses, it is easy to cause a series of related psychological problems such as anxiety and depression. Because the age of the students are suffering from severe acute respiratory syndrome (SARS) is still young. They have not been exposed to such public health incidents (Xiaofei and Dongmei, 2005). This kind of unknown public health incident may exacerbate the negative psychology of college students. Meanwhile, another study showed that the public’s psychological condition has changed in stages with the development of the pandemic (Yaner, 2020). The results of this study showed that this phased change changes with the overall severity of the pandemic. For students in different regions, studies have shown that with the increase in the cumulative number of confirmed cases in the local area (Fang and Bing, 2020), public psychology tends to be tense and negative. Most studies now believe that if the pandemic situation worsens, people’s psychological state will worsen with it. The results of our study were in line with this trend in the first to second stages. On the contrary, with the repeated outbreak of the epidemic in the third stage, the psychological state of the students improved slightly. There may be two reasons for this. First, the three-stage epidemic is not as sudden and severe as when the epidemic first broke out. College students at this time have already experienced the impact of the pandemic and are not easily affected anymore. Second, before August 2021, colleges and universities have implemented effective psychological counseling measures for college students, which can help promptly even when the epidemic worsens. But another survey showed that the level of exposure to COVID-19 in China was negatively associated with mental health problems, which confirmed the “Psychological Typhoon Eye” effect (Zhang et al., 2020). Therefore, when doing psychological counseling for college students, especially during the holidays, the psychological state of students in non-risk areas should not be ignored, and should pay the same attention to students in high-risk areas.

The impact of unhealthy behavior on the mental state should be valued. From Table 5, starting to eat a lot to relieve emotions, smoking and drinking more than usual, and praying for ancestors or gods’ blessings are all affected by the development and changes of the pandemic. In the general population, both increased restricting and binge eating behaviors were reported (Phillipou et al., 2020). Binge eating, alcoholism, and worship are all unhealthy behavioral measures to cope with COVID-19. Studies have shown that binge eating can temporarily improve mood, and individuals may also reduce negative emotions through binge eating (Crowther et al., 2001). Both binge eating and alcoholism can be seen as the result of diverting the individual’s attention to the surrounding environment to escape negative emotions. While worshiping is looking for psychological support to eliminate negative emotions. However, the above irrational behaviors may cause more serious consequences for mental health. Individuals with binge eating reported more stress events, lower tolerance for negative emotions, and greater difficulties in emotional awareness (such as alexithymia, psycho sensory disturbance, etc.) (Zeeck et al., 2011). The present study found that the relationships between fear of COVID-19, changes in psychological state, and unhealthy behavior in Figure 1 were significant. The mediating effect analysis shows that the unhealthy behavior in Figure 1 plays a partially mediating role in fear of COVID-19 and changes in psychological state. Research has shown that the fear of COVID-19 facilitates the development of psychiatric symptoms among those who previously did not experience mental illness (Shigemura et al., 2020). Some studies revealed that preventive health behavior has a positive relationship with fear of COVID-19 but a negative relationship with psychological distress (Olapegba et al., 2021). The different feelings about the source of pandemic fear are due to the different degree and breadth of students’ cognition of it. According to SCRC theory, individuals will have psychological and emotional reactions after cognitive assessment and then adopt crisis response behaviors. However, the mediating effect results of this study show that cognition indirectly affects psychological changes through behavioral changes. In general, according to the results of this research, we believe that the relationship between recognition, psychological, and behavioral changes is not static, but a dynamic and mutually influencing relationship between them.

This study has several strengths. We have collected a large and geographically diverse sample during the COVID-19 pandemic. Importantly, the current research about college students’ mental health during the COVID-19 pandemic is mainly from a static period at present, this study is an initial effort to explore changes in college students’ psychological status during pandemic normalization. Our research is not only critical to our understanding and handling of the psychological consequences of college students but also to help predict future mental states. Based on previous research on infectious diseases (such as SARS), the research makes recommendations for colleges and universities to prevent college students from developing psychological problems. We investigated the effect of cognition on the psychological state of college students, we should improve college students’ awareness of the pandemic while simultaneously avoiding information overload. In addition, the mediating role of behavioral changes between recognition and mental state changes also reminds us that students with abnormal behaviors cannot be ignored.

However, the current study has several limitations. First, although the current study recruited a wide range of college students for the survey, the sample size of the questionnaire did not cover all regions and major cities across the country. Therefore, the conclusions cannot accurately infer differences in demographic variables. Besides, all the participants are from China and cannot represent all the countries and cultures. Future research can be conducted on a larger scale and get more comprehensive data. Second, the open recruitment method via the internet also has its drawbacks. Our sample most are for medical students (68%). Potentially, Medical students may be more willing to participate in the study. The lower proportion of negative emotions such as anxiety, and depression among medical students may interfere with the overall mental state score. Additionally, the major makeup and recruitment method may lead to findings not being representative of the larger Chinese university student population. Third, given the study’s mediating effect, the relationship between cognitive degree, mental state changes, and behavioral changes cannot be ascertained. What this study can determine is that the cognitive degree can have a certain impact on the psychological state of college students by changing their behavior, this could be due to those who have lower recognition of the pandemic being more likely to engage in actions as a coping. But whether behavior can directly affect the mental state changes or vice versa, is still unclear. Future studies may explore these associations. The generalization of conclusions has yet to be tested through more investigations. Finally, our discussion of influencing factors is relatively simple. Some studies have pointed out that the COVID-19 pandemic has affected various aspects such as the social environment and economy to varying degrees (Wang and Su, 2020; Wang and Zhang, 2021). How will these indirect factors affect the mental health of college students? This is what we will consider in future research. These interesting problems mentioned above deserve further study.

## Conclusion

With the effective control of the pandemic, the mental state of the students showed a slight improvement in the environment of sporadic cases. Behavior has a partial mediating effect between the source of fear and psychological changes. Correct behavior guidance can effectively reduce the psychological changes caused by college students’ fear. This is of great significance for universities to take correct intervention measures for the problems of college students’ psychological state during the COVID-19 pandemic.

## Data Availability Statement

The raw data supporting the conclusions of this article will be made available by the authors, without undue reservation.

## Ethics Statement

The studies involving human participants were reviewed and approved by the Asia University Medical Research Ethics Committee. Written informed consent for participation was not required for this study in accordance with the National Legislation and the Institutional Requirements.

## Author Contributions

CL, JT, and WH participated in the study design. CS, JT, XZ, BS, and EB carried out the surveys. CS and CL performed the statistical analysis. XZ, EB, and WH contributed to the analysis. JT, XZ, and CL wrote the manuscript. All authors read and approved the final manuscript.

## Conflict of Interest

The authors declare that the research was conducted in the absence of any commercial or financial relationships that could be construed as a potential conflict of interest.

## Publisher’s Note

All claims expressed in this article are solely those of the authors and do not necessarily represent those of their affiliated organizations, or those of the publisher, the editors and the reviewers. Any product that may be evaluated in this article, or claim that may be made by its manufacturer, is not guaranteed or endorsed by the publisher.
