# Supplementary material for: Research of the Changes in the Psychological Status of Chinese University Students and the Influencing Factors During the COVID-19 Pandemic
Source: Front Psychol. 2022 May 31;13:891778. doi: 10.3389/fpsyg.2022.891778 (PMC9197466; doi:10.3389/fpsyg.2022.891778)
Supplement: Supplementary file 1 [file Table_1.DOC]

Supplementary Material

# Supplementary Tables

## Supplementary Tables

**Supplementary Table1.** P Multicollinearity check for data in Hierarchical multiple regression analysis (VIF)

| Variable | Dependent variable: Total mental state score | | |
| --- | --- | --- | --- |
|  | Phase 1 | Phase 2 | Phase 3 |
| Controlled variable: |  |  |  |
| Gender | 1.038 | 1.059 | 1.023 |
| Ethnicity | 1.107 | 1.028 | 1.026 |
| Grade | 1.021 | 1.137 | 1.045 |
| Major | 1.108 | 1.107 | 1.036 |
| Is it a student leader | 1.149 | 1.033 | 1.036 |
| Independent variable: |  |  |  |
| Recognition to the pandemic | 1.044 | 1.034 | 1.023 |
| Fear of a highly contagious virus | 1.581 | 1.432 | 1.557 |
| Fear of no specific treatment | 1.536 | 1.560 | 1.836 |
| Fear of the emergence of sporadic cases | 1.597 | 1.519 | 1.719 |
